# Optimization of synchrotron radiation parameters using swarm intelligence and evolutionary algorithms

**DOI:** 10.1107/S1600577524000717

**Published:** 2024-02-22

**Authors:** Adnan Sahin Karaca, Erkan Bostanci, Didem Ketenoglu, Manuel Harder, Ali Can Canbay, Bora Ketenoglu, Engin Eren, Ayhan Aydin, Zhong Yin, Mehmet Serdar Guzel, Michael Martins

**Affiliations:** a Department of Computer Engineering, Ankara University, 06830 Ankara, Türkiye; b Department of Engineering Physics, Ankara University, 06100 Ankara, Türkiye; c European XFEL GmbH, Schenefeld, Germany; d Department of Physics, Hamburg University, 22761 Hamburg, Germany; e Department of Physics, Ankara University, 06830 Ankara, Türkiye; f Deutsches Elektronen-Synchrotron (DESY), 22607 Hamburg, Germany; g International Center for Synchrotron Radiation Innovation Smart (SRIS), Tohoku University, Sendai 980-8577, Japan; h Institute of Experimental Physics, Hamburg University, 22607 Hamburg, Germany; i Center for Free-Electron Laser Science (CFEL), 22607 Hamburg, Germany; University of Tokyo, Japan

**Keywords:** synchrotron beamlines, KB mirrors, Be compound refractive lenses, swarm intelligence, evolutionary algorithms, multi-objective optimization

## Abstract

Alignment of each optical element at a synchrotron beamline takes days, even weeks, for each experiment costing valuable beam time. Evolutionary algorithms (EAs), efficient heuristic search methods based on Darwinian evolution, can be utilized for multi-objective optimization problems in different application areas. In this study, the flux and spot size of a synchrotron beam are optimized for two different experimental setups including optical elements such as lenses and mirrors. Calculations were carried out with the *X-ray Tracer* beamline simulator using swarm intelligence (SI) algorithms and for comparison the same setups were optimized with EAs. The EAs and SI algorithms used in this study for two different experimental setups are the Genetic Algorithm (GA), Non-dominated Sorting Genetic Algorithm II (NSGA-II), Particle Swarm Optimization (PSO) and Artificial Bee Colony (ABC). While one of the algorithms optimizes the lens position, the other focuses on optimizing the focal distances of Kirkpatrick–Baez mirrors. First, mono-objective evolutionary algorithms were used and the spot size or flux values checked separately. After comparison of mono-objective algorithms, the multi-objective evolutionary algorithm NSGA-II was run for both objectives – minimum spot size and maximum flux. Every algorithm configuration was run several times for Monte Carlo simulations since these processes generate random solutions and the simulator also produces solutions that are stochastic. The results show that the PSO algorithm gives the best values over all setups.

## Abstract 2

Different algorithms were tested on a synchrotron beamline. Results show that the swarm intelligence algorithm and the particle swarm optimization algorithm perform better than previous algorithms.

## 1. Introduction

Synchrotron beamlines need continuous realignment of optical elements, since noise and vibrations cannot be ignored for accurate measurements. Additionally, different requirements for a variety of experiments call for different experimental setups, thus realignment is crucial. Beamlines are like electric circuits that are connected in series – thus any failing part prevents the synchrotron beam from hitting the sample; even slight changes in the angle or position of an optical element may cause significant effects. The current study focuses on optimizing the beamline optics, which play a vital role in measurement quality. Beamline optics are utilized for collimating, focusing and monochromatizing the beam with the required properties from source to sample (Hart, 1996 ▸; Ketenoglu, 2019 ▸). Beam characteristics such as flux, photon beam energy (wavelength), energy bandwidth (monochromaticity), spot size and polarization are adjusted at beamlines depending on the dedicated experiment.

In the earliest studies, optimization was carried out on storage rings (Zisman, 1987 ▸); software written in Fortran was developed to enable studies on the storage ring parameters. Circumference, momentum compaction factor, natural emittance and damping times are machine parameters whereas energy, intensity, bunch length and momentum spread are the beam parameters, and frequency, voltage and higher-order cavity modes are the radiofrequency system parameters that were intended to be optimized with this program. This study was implemented on the LBL (now ALS) synchrotron facility in California, USA. Just as optimizations were used on existing systems, there were studies that made use of optimization techniques in the design phase (Shimano *et al.*, 1992 ▸). Compact storage rings and beamlines were designed by the ray-tracing method and flux was used as an objective function. Optimization studies were carried out on particle accelerator facilities as well, similar to synchrotron facilities (Catani, 1997 ▸). Evolutionary strategies were used on coils for optimization using specifications of the LISA facility in Italy. Expert systems were also used on an earlier study for intelligent decision making (Svensson & Pugliese, 1998 ▸). The alignment problem was solved by using a set of rules taken from beamline technicians. Flux was optimized with genetic-based algorithms on the SSLS facility XAFCA beamline in Singapore. The orientation of optical elements was adjusted by six-axes motors for maximum flux (Xi *et al.*, 2015 ▸). In a follow-up study at the same facility, a genetic algorithm was used with a differential evolution algorithm with the same set of parameters. It was suggested in this study that the genetic algorithm gives results with less generation but more working time (Xi *et al.*, 2017 ▸). In a similar approach, particle swarm optimization and the genetic algorithm was used on an ion accelerator facility and transmission maximization was selected as the objective function (Appel *et al.*, 2017 ▸). The study concluded that other optimization techniques should be used and beam size and position should be included. The multi-objective NSGA-II algorithm was used on the SIBERIA-2 facility in Russia; horizontal emittance and dynamic aperture were selected as objective functions by Korchuganov *et al.* (2018 ▸). While minimizing the objectives it was aimed to have a fixed magnetic elements geometry, elements position and feed circuit. In 2019, a hard X-ray free-electron laser (FEL) was optimized using the NSGA-III algorithm (Ketenoglu, Bostanci *et al.* 2019 ▸). Saturation power, the Pierce parameter and saturation length parameters were optimized. The NSGA-II algorithm was used on radiotherapy applications using collimating magnets on a proton accelerator, and the Pareto front was found for clinical requirements (Liu *et al.*, 2020 ▸). Aydin *et al.* (2020 ▸) made use of multi-objective optimization and evolutionary algorithms at the TARLA facility in Turkey. They used NSGA-II, NSGA-III, VEGA and RVEA algorithms on beam position monitors and optimized the signal-to-noise ratio using the antenna radius, gap and thickness as well as the beam pipe diameter. It was found that NSGA-II is the best algorithm out of all four. The latest study performed on undulators for gap optimization by the authors of this paper (Ketenoglu *et al.*, 2023 ▸) was carried out using the VEGA, GA, DE, PAES, ɛ-MOEA, NSGAII, GDE3 and NSGA-III algorithms. In the optimization process, undulator gap and number of photons are taken as input parameters while objective functions (*i.e.* fitness functions) are taken as brilliance and λ_ph_. NSGA-II and VEGA yielded peak brilliance while NSGA-III provided minimum brilliance. Zhang *et al.* (2023 ▸) used the NSGA-II algorithm at beamline ID17 of the European Synchrotron Radiation Facility (ESRF) with energy and dose rate as objectives. They successfully optimized both objectives with an energy increase of 7% and dose rate of 20%. An optimal solution set can be obtained within 30 generations. The *SHADOW* simulator was used for this task.

Simulators, or simulation software, have often been utilized in this field. The *SHADOW* ray-tracing simulator was used for an optical system optimization study by Li *et al.* (1993 ▸). Three optical elements – reflection mirror, silicon filter and beryllium windows – were used in the simulations. Technical specifications were obtained from the SSRC facility. Desired results were obtained for a lithography experiment. In another study, the *SHADOW* simulator was used for designing an optimum beamline on the AMOS beamline at the INDUS-2 facility (Das *et al.*, 2014 ▸). Similar to *SHADOW*, another simulator named *SRW* was developed and validated at the ESRF and SOLEIL facilities on infrared beamlines (Chubar *et al.*, 2007 ▸). *SRW* was again used on I13 beamline at the Diamond Light Source facility. Optical elements were controlled for optimization and genetic algorithm parameters were 50 generation and 100 population (Taheri *et al.*, 2019 ▸). The *SIMPLEX* simulator was used on a FEL for optimizing undulator parameters such as gain length, saturation power and saturation length using numerical calculations (Ketenoglu, Aydin & Yavas, 2019 ▸). *X-Ray Tracer* (*XRT*) was used at the Canadian Light Source facility (Heredia *et al.*, 2019 ▸). Optical element combinations were tested on the simulator and it was found that V-shaped apertures are needed for their experimental setup. A simulator named *SYRIS* was developed and validated at DESY PETRA III (Otte *et al.*, 2019 ▸), intended to be used for experiment preparation, instrument operation and analysis benchmarks.

From the literature, one can see that simulators have been widely used on beamlines and have proven to have benefits on optimization of the designing and readjustment phases. EAs were used and results were tested on existing beamlines. Almost all of the studies used different setups and parameters. Thus a robust optimization software should be tested on different beamlines with different optical elements.

For this study Swarm Intelligence (SI) algorithms were tested and then compared with EAs. Simulations were carried out in mono- and multi-objective modes. This work contains the Genetic Algorithm (GA), Non-dominated Sorting Genetic Algorithm-II (NSGA-II) for EA and Particle Swarm Optimization (PSO) and Artificial Bee Colony (ABC) for SI. ABC had never been used on this type of problem, while PSO had only been used on a remotely related experimental setup and objective functions. Maximum flux and minimum spot size were selected as the objective functions owing to its use on a variety of applications. These objective functions were tested on two different experimental setups; one of them focuses on a single beryllium compound refractive lens and the other focuses on a mirror pair – the Kirkpatrick–Baez (KB) mirror. KB mirrors are used at the SOLEIL, SLS (Mercere *et al.*, 2007 ▸) and DESY facilities (Ketenoglu *et al.*, 2015 ▸, 2018 ▸). KB mirrors with good focusing ability were used and the beam was focused to the micrometre level. Beamline setups were provided from DESY PETRA III scientists.

The rest of the paper is laid out as follows. In Section 2, the optimization concepts and algorithms are explained briefly. In Section 3, simulator results are presented – an experimental setup on simulator is included in this chapter. The conclusions are drawn in Section 4.

## 2. Optimization algorithms

Optimization is the selection of best elements with regard to some criterion from some set of available alternatives. Many real life problems can consist of one or more criteria called objectives. In our problem we are going to minimize spot size and maximize flux. When there are more than one conflicting objectives, multi-objective methods are used,

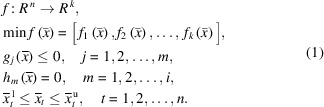

Here, *n* is the number of decision variables, *k* is the number of objective functions, *m* is the number of inequality constraint functions, *i* is the number of equality constraint functions, *g* and *h* are constraint functions, and *x* ^−l^ and *x* ^−u^ are lower and upper boundaries of the decision variables (Marler & Arora, 2004 ▸).

**Figure 1:**
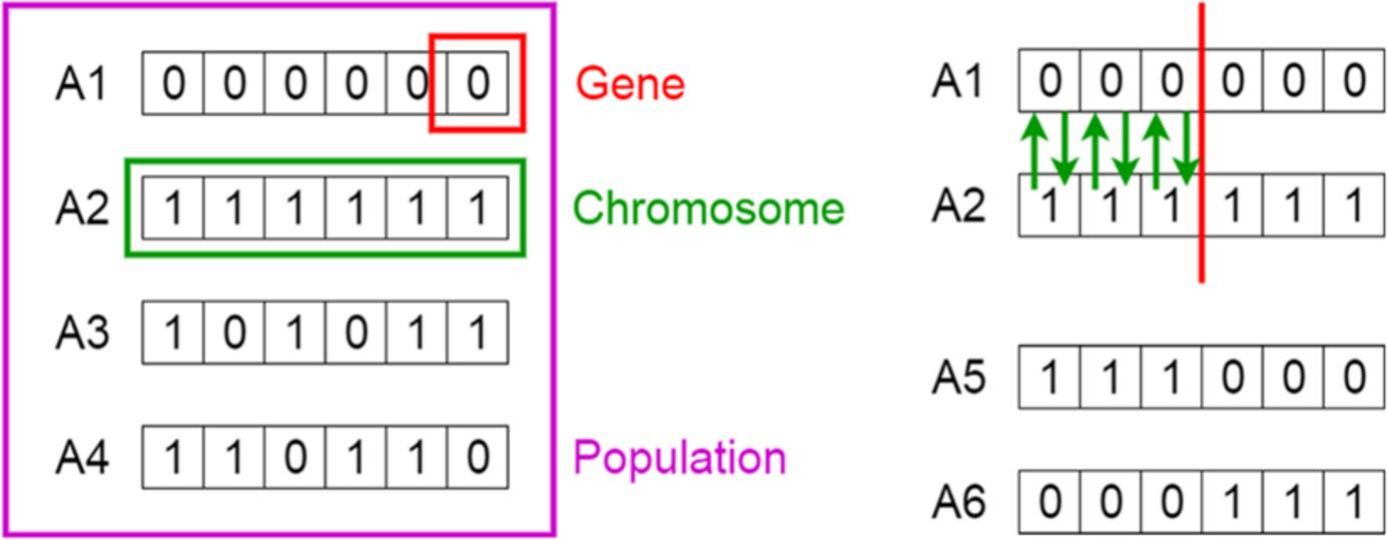
EA terminology.

EAs imitate evolution theory. In its core concepts it contains reproduction, mutation, recombination and selection of candidate solutions for an optimization problem. The terms were borrowed from biology, as can be seen in Fig. 1 ▸. Here, an individual is every coded solution. Population is the pool of individuals. Selection is choosing the fitter individuals. A gene is one bit of information in a coded individual. Mutation is changing only one gene of a chromosome. A chromosome is every gene of the individual. Crossover is exchanging some genes between two chromosomes. Generation is the new population after the best individuals are selected.

**Figure 2:**
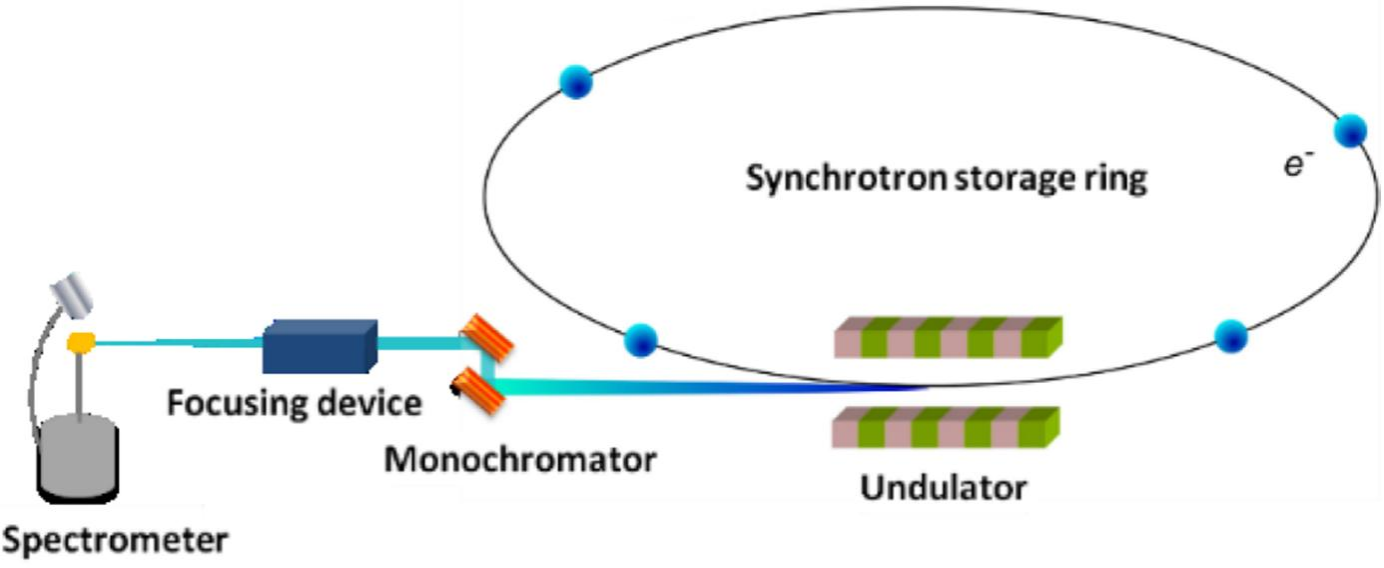
Synchrotron beamline (Ketenoglu *et al.*, 2019 ▸).

For our problem we want to change the focusing device position for the objective of maximum flux and minimum spot size in Fig. 2 ▸. The fitness criteria for our optimization problem are minimum spot sizes and maximum flux values. Our decision variable becomes the position value in millimetres.

**Figure 3:**
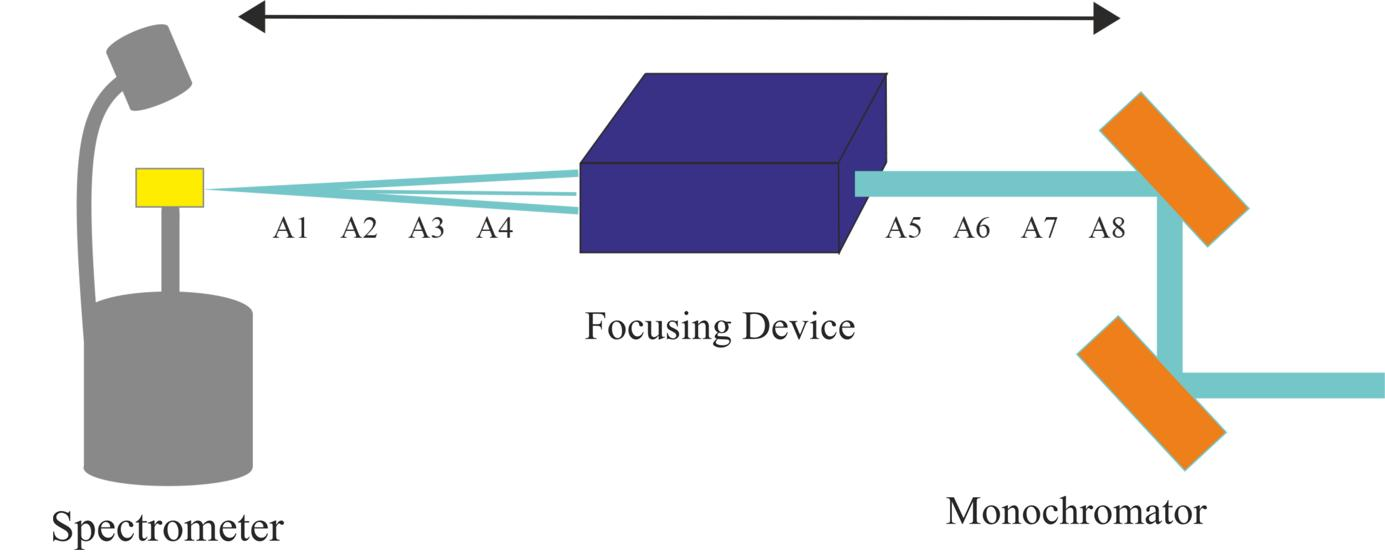
Focusing device shifting.

For the first generation the system creates random uniformly distributed distance values as in Fig. 3 ▸. Each distance value is coded in binary and becomes an individual. In this example population the size is eight. Some individuals change through crossover and mutation. Then their fitness (spot size) is measured using the simulation software *XRT* by shifting the focusing device from the position A1 to position A8. This process is repeated until the best values or values that are good enough were found.

One of the oldest and most tested evolutionary algorithms is the genetic algorithm (GA). The GA is a very basic form of the EA. Thus every concept is valid for GA. Algorithm performance is largely impacted by its parameters such as population and generation numbers, mutation and crossover probabilities (Goldberg, 1989 ▸).

**Figure 4:**
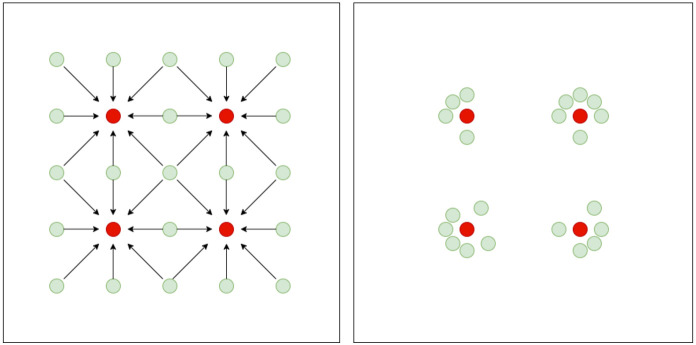
Swarm behavior.

SI mimics the natural behavior of insects. In PSO, compared with GA, we use particles instead of chromosomes. Particles (candidate solutions) started randomly and their fitness is measured similar to GA. Best particles are selected and then neighboring particles change position towards these particles as in Fig. 4 ▸. This process repeats until a user-defined convergence criterion is met (Kennedy & Eberhart, 1995 ▸).

**Figure 5:**
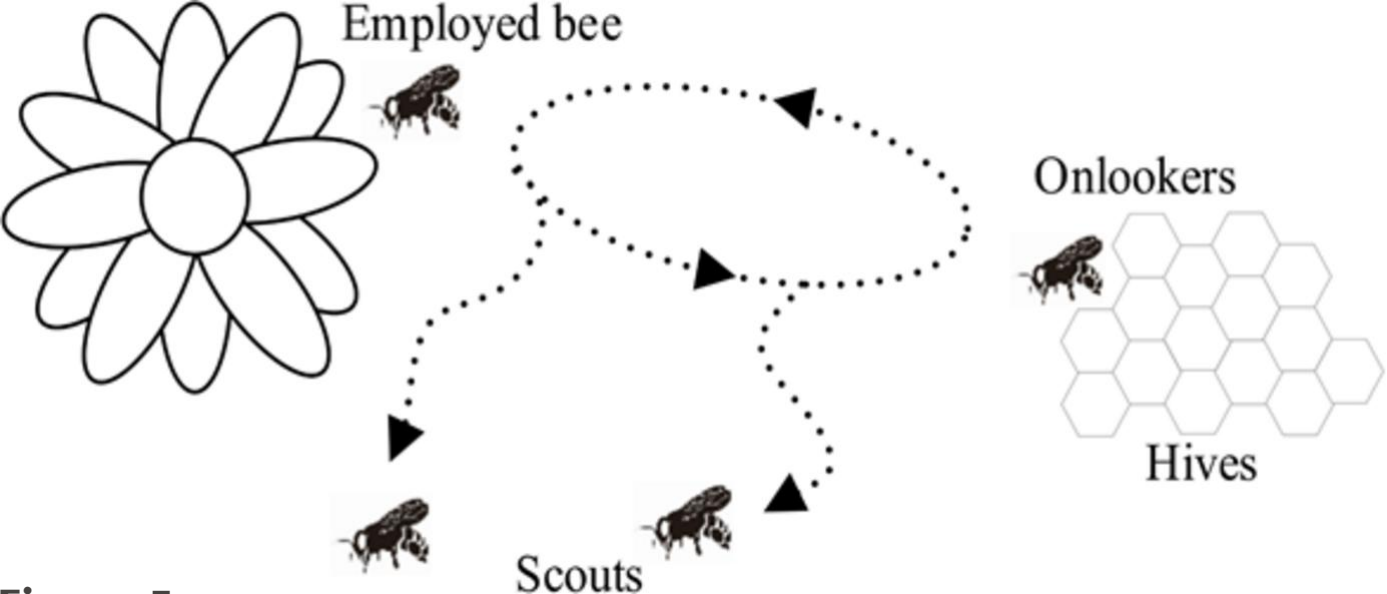
Bee colony.

The ABC algorithm is an SI algorithm similar to PSO. This uses a bee analogy. In this algorithm the setting parameters are colony size and maximum cycle. The individuals are called food positions and their fitness is evaluated by bee movements. Scout bees choose a food source randomly and evaluate these sources as in Fig. 5 ▸. Employed bees and onlookers use their experience. The better the food source, the more vigorous the bee dance, thus attracting other bees. If a better source is discovered, the previous source is forgotten. This process is repeated until the cycle number is reached or a convergence criterion is met which is set by the user (Karaboga, 2010 ▸).

**Figure 6:**
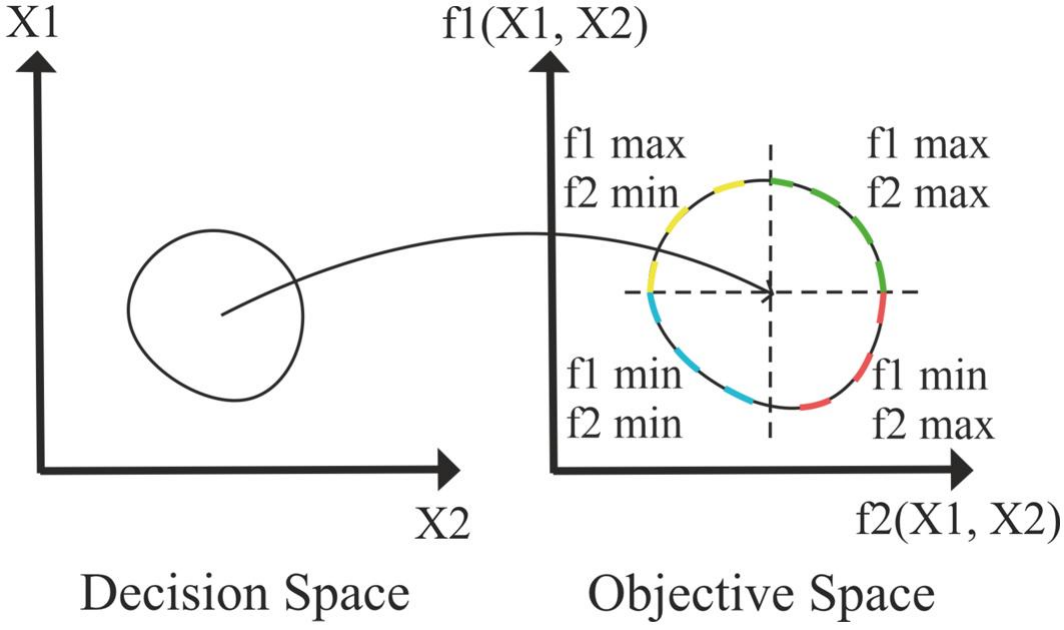
Transition from decision space to objective space by the objective function.

In single objective optimization there is only one minimum or maximum. In multi-objective optimization we try to find the Pareto front that contains the best set of solutions. In Fig. 6 ▸, colored dashed lines are called the Pareto front. Since we can find either a minimum or a maximum of an objective, different regions are found accordingly. Since one objective is minimized and the other is maximized, the red Pareto front is the solution.

**Figure 7:**
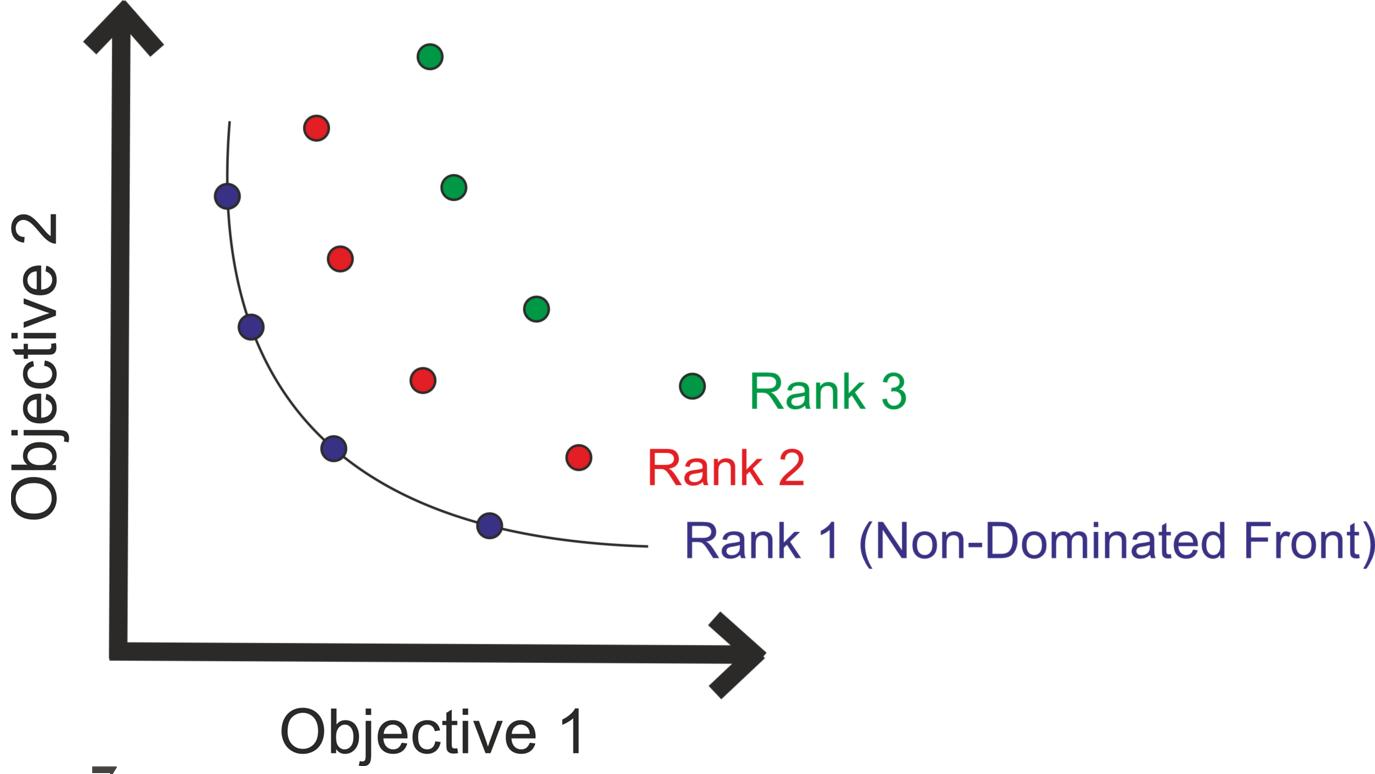
Ranking of the solutions.

A set of results is non-dominated if two values have a negative covariance relationship meaning one objective value gets better while the other objective value gets worse (Fig. 7 ▸). In NSGA-II we use these non-dominating relations.

**Figure 8:**
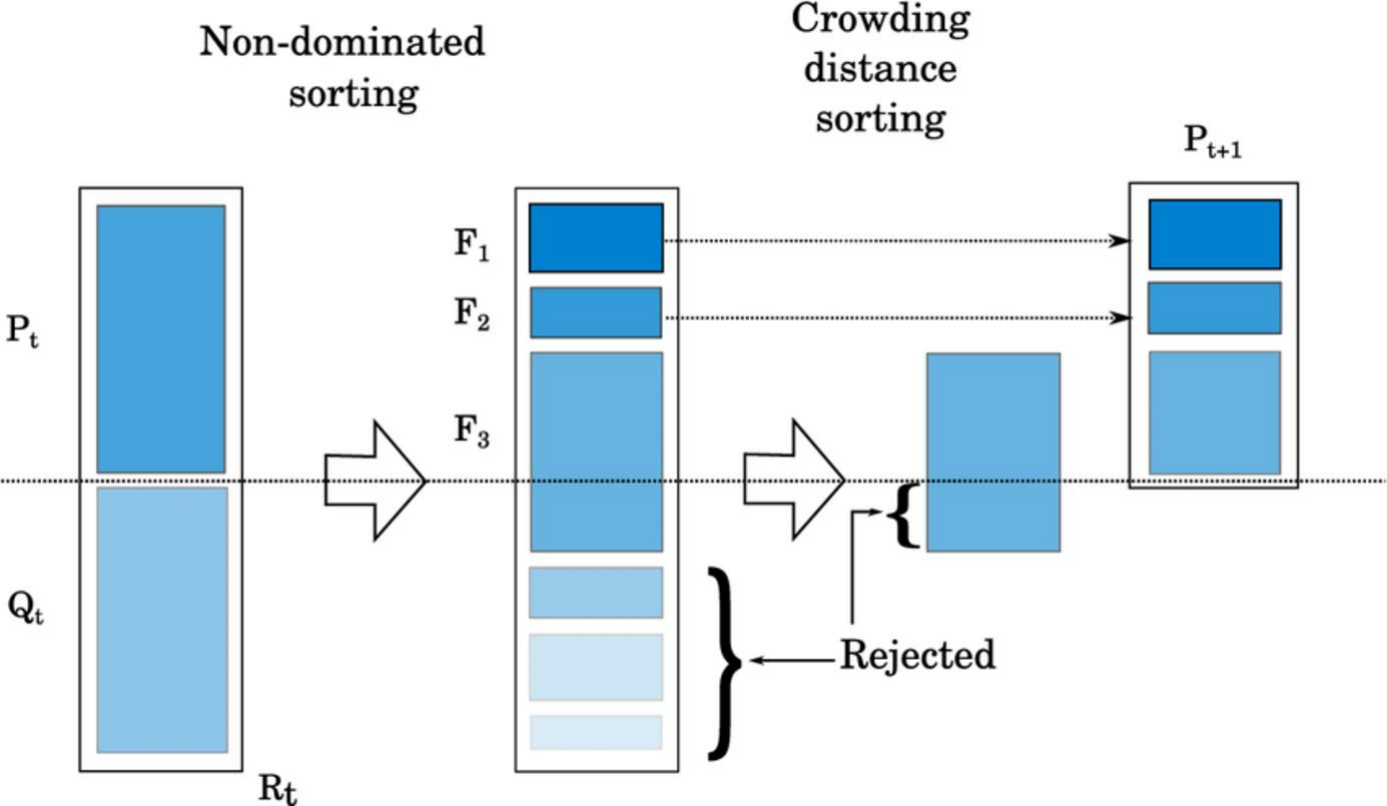
NSGA-II schematic.

In Fig. 8 ▸, at first the child population Q_t_ is produced from the parent P_t_ population using conventional genetic operators. Then the two populations are merged. Solutions are ranked depending on their distance to the Pareto front. The crowding distance is measured from these ranked points. Then a crowding comparison is used to select the next generation. The points with the highest rank and lowest crowding distance at the same rank are assumed to be non-dominated and consist of next-generation individuals. This process is repeated until the convergence criterion is met (Deb *et al.*, 2002 ▸).

There are two types of algorithms in this study. GA and NSGA-II are EAs whereas PSO and ABC are SIs. All four of the algorithms use random processes which help the blind search in an unknown search space. GA uses mutation and crossover at the decision variables which are coded in binary. Selecting only the best solutions might stop the algorithm at the local minima or maxima points. Thus these genetic operators help to skip these points and search undiscovered areas. This is especially helpful since most of the search space provides zero value for the objective function. On the other hand a strong feature of NSGA-II is multi-objective optimization. Since the first part of the algorithm is classic GA, this algorithm adds unnecessary complexity on single objective optimization while trying to rank solutions depending on their Pareto front which is a global extremum on a single mode and separation of these solutions using the crowding distance. SI algorithms such as PSO and ABC use global best and local best as part of their search strategy. So they do not solely rely on better half and random jumps. Instead they divide the search space into regions and create local bests. The best of the local bests is the global best. So in the next generation the rest of the solutions change their position towards these best values. If the value is better, the position change is faster. The difference between PSO and ABC is that in PSO all of the swarm behaves the same while in ABC the swarm is divided and given different roles such as scouts, onlookers and employed bees. This means that we have more parameters to adjust which creates complexity. When the number of parameters increases, the algorithms require additional study for parameters other than the default ones. Given this information, SI algorithms are expected to perform better since the swarm searches the space with prior knowledge and semi random jumps since beamline optics work in series and function only at certain positions and angles.

For this study the iteration/generation number is fixed for all the algorithms and is 100. The population/particle number is 20. The same set of parameters were used for comparison as much as possible.

## 3. Experiments on simulation

*XRT* is a ray tracer simulator that enables users to tweak around the parameters of different source types such as undulators and wigglers and optical elements such as slits, lenses, mirrors *etc*. (Klementiev & Chernikov, 2014 ▸). *XRT* is written in Python language. There is a module called *XrtQook* for visualization that helps to see changes in beam geometry and the sample. For reading the results we must also add screens to the experimental setup.

**Figure 9:**
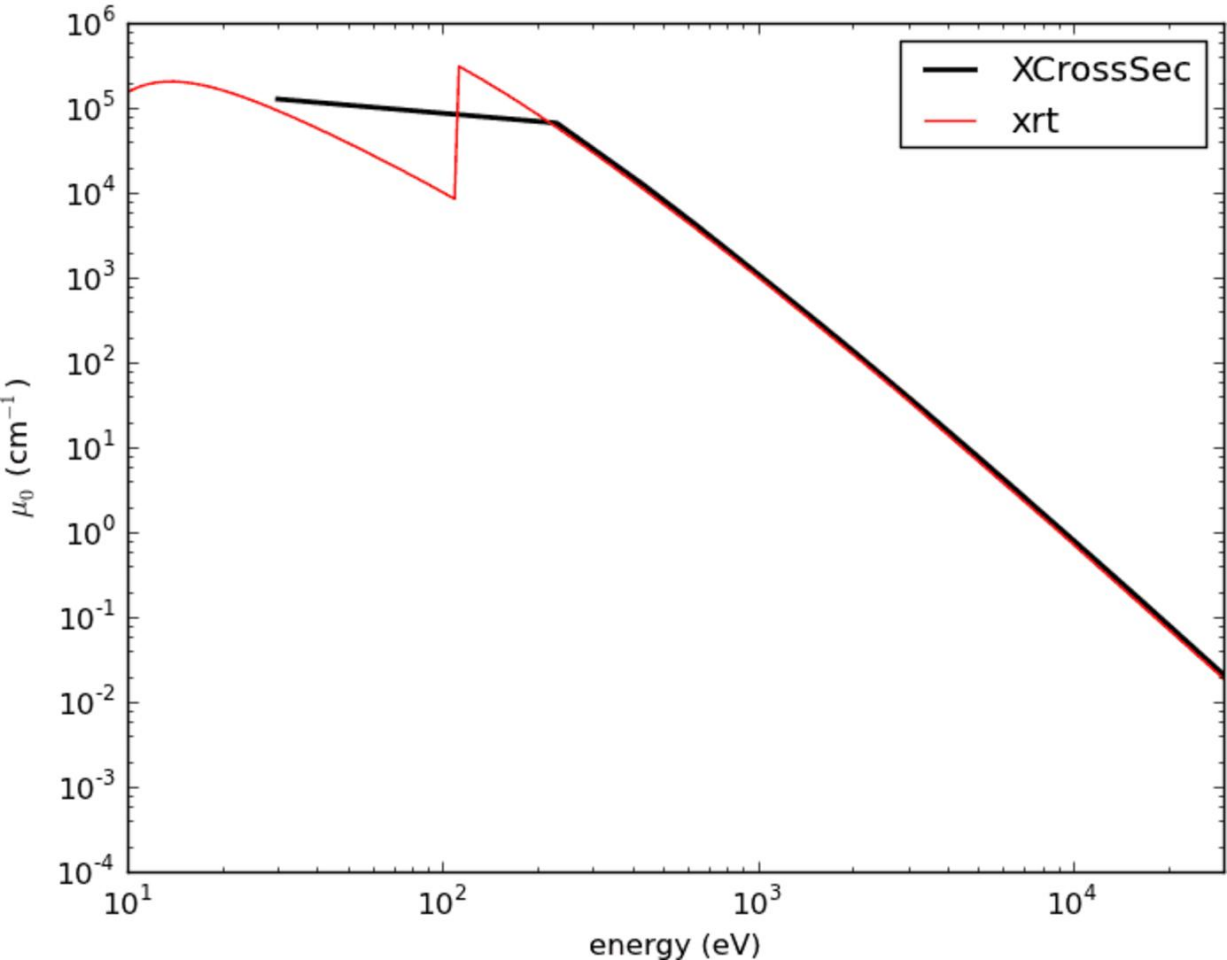
Be absorption as a function of energy.

We used geometric source for calculations. Apart from the parameters in Table 1 ▸, the rest of the parameters were left as default. The beam is elliptical in shape so the results emerge in elliptical form. For setups including a lens we used a beryllium compound refractive lens with a density of 1.85 g cm^−1^ at a 33 m-long beamline with focus 0.1 mm, number of lenses 6 and thickness 0.1 mm with a *z*-axis limit of 1 mm, so the overall thickness is 1.1 mm. The rest of the parameters were left as default. We stationed a screen to read the parameters at a sample position of 33 m. Absorption of Be changes with energy, as can be seen in Fig. 9 ▸.

**Figure 10:**
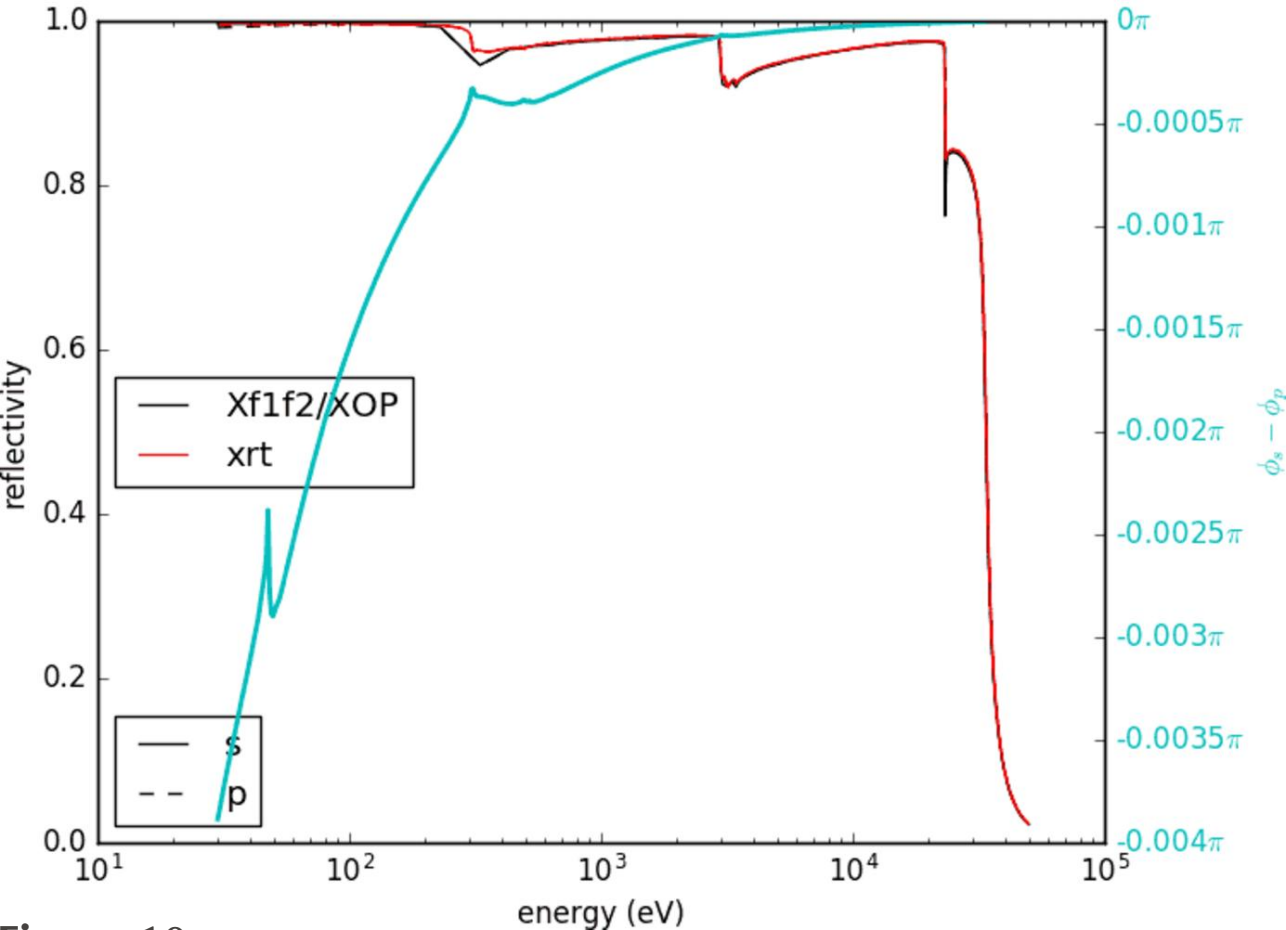
Rhodium reflectivity as a function of energy.

The position of the lens was changed between 10 m and 33 m by the algorithm to find the optimum solution. A double-crystal monochromator (DCM) was stationed 20 m away from the source. For setups including KB mirrors, a pair of rhodium mirrors of density 12.38 g cm^−1^ were used at a 70 m-long beamline where the screen is at the end. The reflectivity of rhodium changes depending on the energy, as can be seen in Fig. 10 ▸.

**Figure 11:**
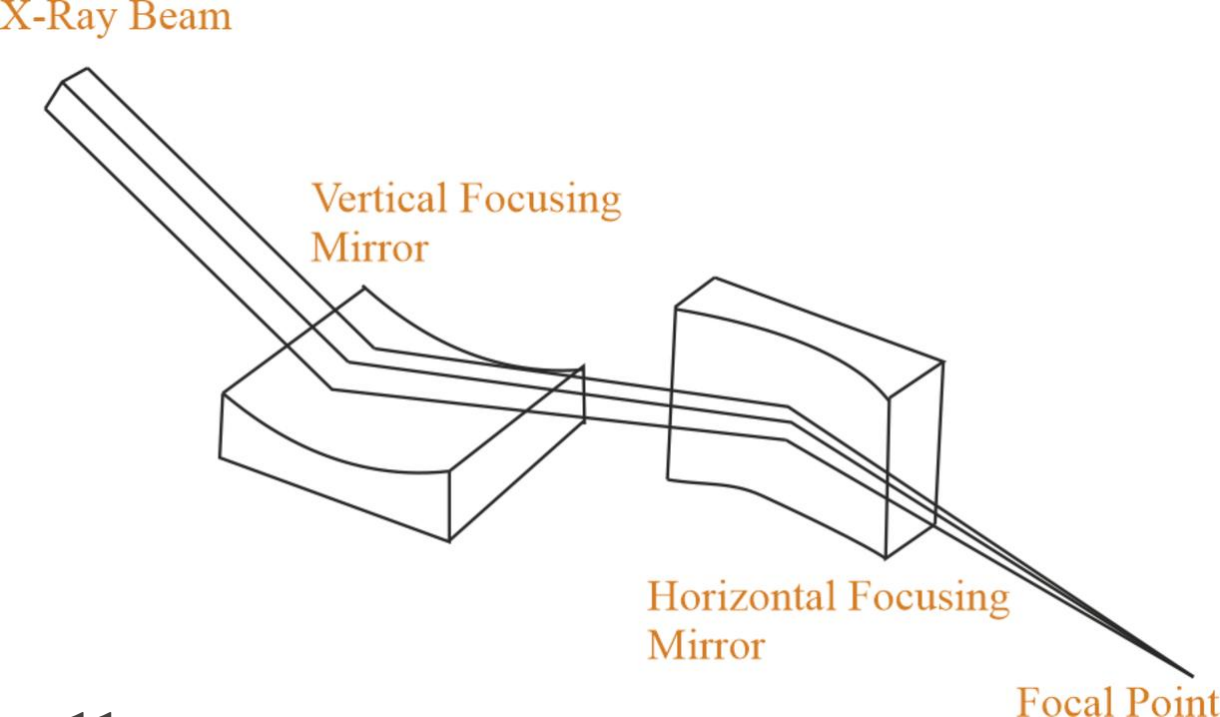
Schematic representation of KB mirrors.

There is a DCM at 48 m away from the source and after that there is a pair of mirrors at 68 m (Fig. 11 ▸). The distance between the KB mirrors is 1 m. While one of the mirrors focuses vertically, the other mirror focuses horizontally, thus the mirrors are in a perpendicular orientation.

As can be seen in Fig. 11 ▸, flat mirrors are bent to create a curvature that changes the focus depending on the bending rate. So the radius of curvature is a decision variable calculated by the simulator. Thus for this setup we are going to change the focal distance and optimize flux and spot size. The sizes of the mirrors were adjusted depending on the source size – for this experiment it is 0.145 m × 0.011 m. The pitch of the first mirror is 0.005 and 0.003. The roll value of the first mirror is −1.571. The two parameters in the *XRT* simulator are *p* and *q*.

**Figure 12:**
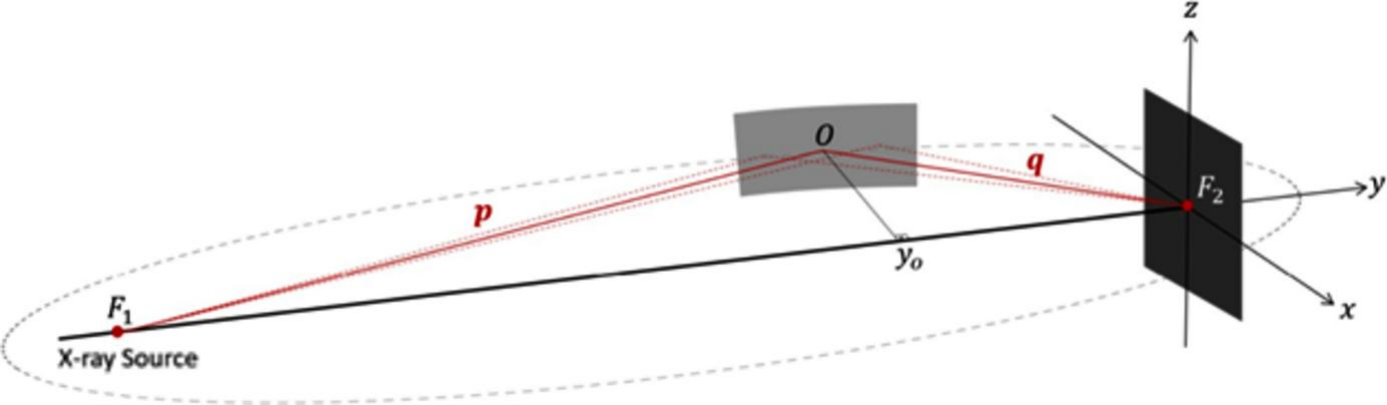
Parameters of one mirror.

As can be seen in Fig. 12 ▸, the *p* value is the defocal distance which is roughly the distance from the source to the mirror. Since there are two mirrors in this setup, there are two *p* and *q* values which we call *p*1, *p*2 and *q*1, *q*2, respectively. Focal points of the mirrors are adjustable and not dependent on their position since the mirrors are foldable instruments. As can be seen in Fig. 12 ▸, the *q* value is the focal distance of the mirror and the *p*1 and *p*2 values can be fixed at the distance between the source and the mirror. The simulator takes care of the bending and we can directly set the focal distance of *q*. So the boundaries of the focal distances are between 0 m and 68 m for the first mirror and between 0 m and 69 m for the second mirror. Since *XRT* contains random processes, we run the algorithms three times and take the average, known as Monte Carlo simulation.

All the optical elements and distances were obtained from the beamline operator at DESY PETRA III.

### 3.1. Mono-objective lens position optimization

**Figure 13:**
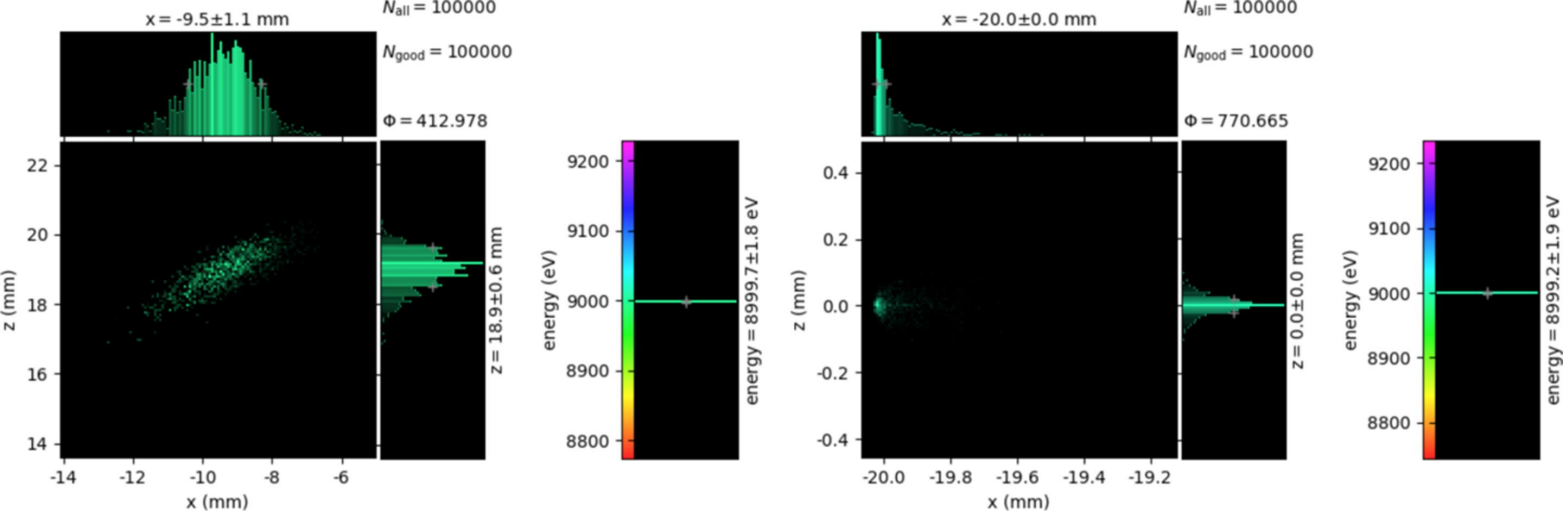
First result for minimum spot size.

*XRT* was run with mono-objective algorithms to observe system and objective function behavior. For illustration purposes the spot size is chosen as the objective. After the algorithm run, as in Fig. 13 ▸, the scatter pattern changes and gives a better spot size and focusing. In Fig. 13 ▸, four algorithms optimized the same setup. NSGA-II is normally a multi-objective algorithm but it has the option to optimize only one objective as well, thus we include it here.

**Figure 14:**
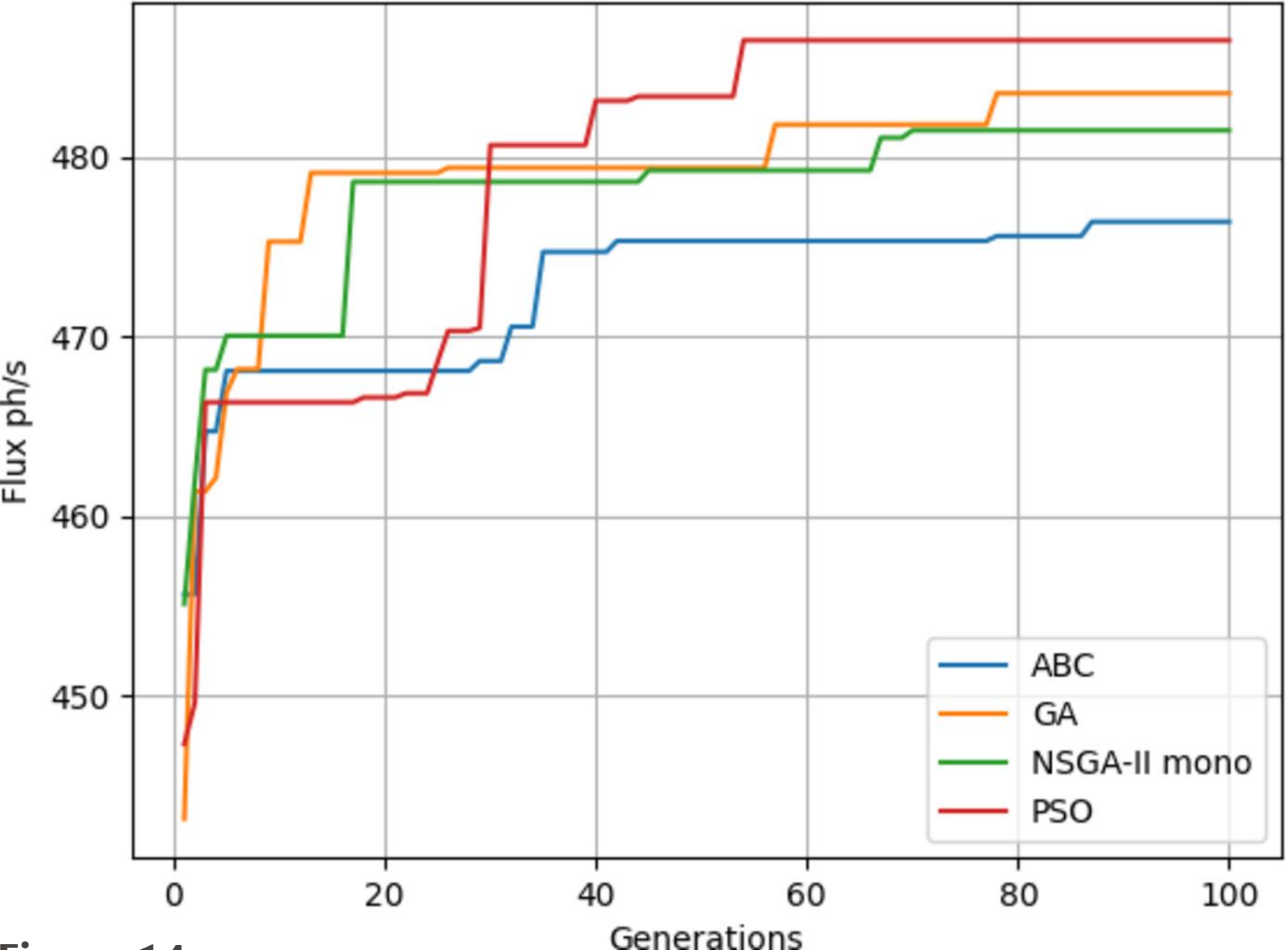
Maximum flux results.

In Table 2 ▸ the results from Fig. 14 ▸ can be seen. On average, the PSO algorithm gives the best results. Distance results are given in millimetres since the simulation takes millimetre values for distances. The best result is highlighted in bold font.

**Figure 15:**
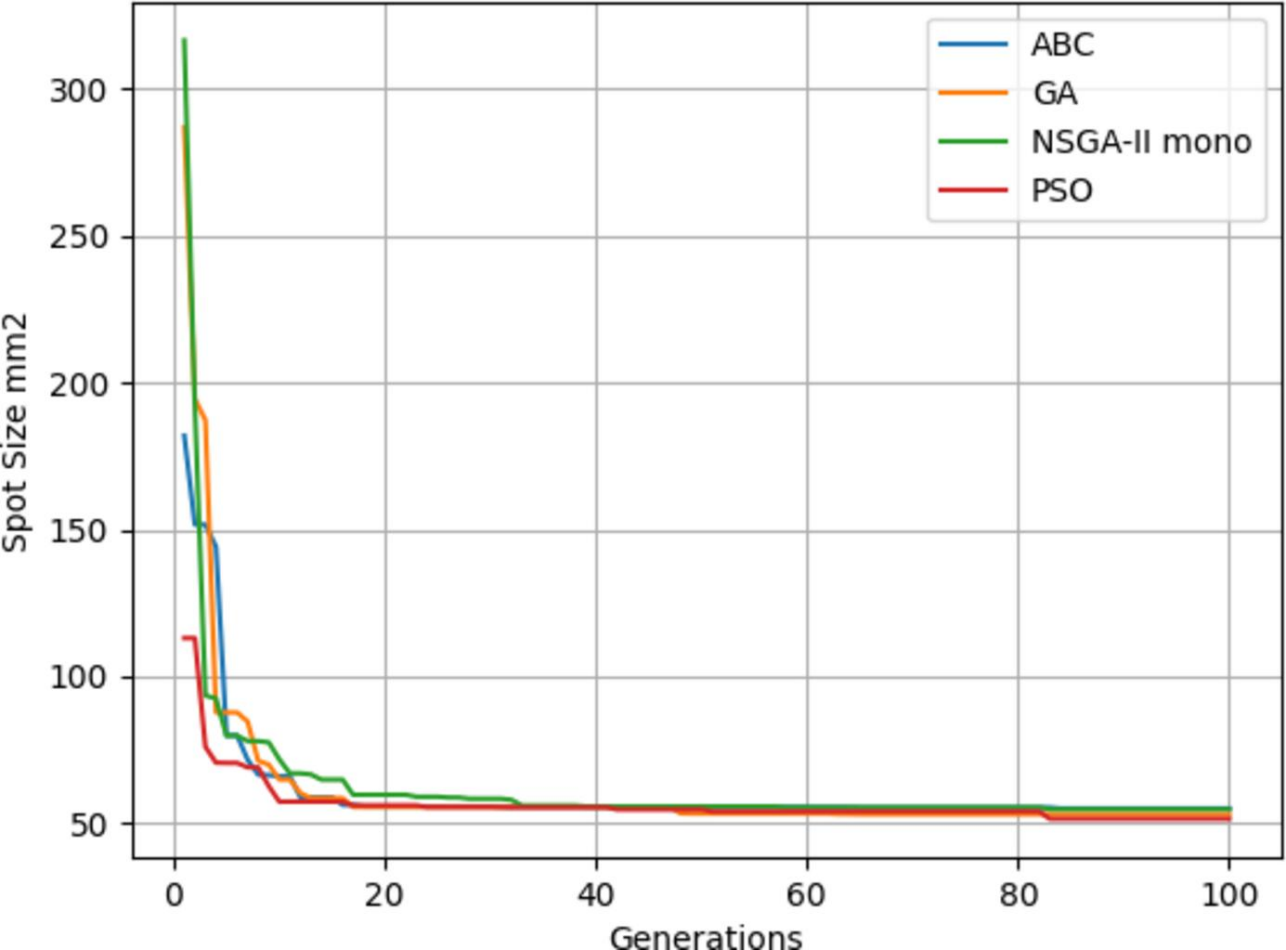
Minimum spot size results.

Fig. 15 ▸ shows the results of four algorithms with the objective of minimum spot size – these are tabulated in Table 3 ▸. On average the PSO algorithm gives the best results. Spot size results are given in mm^2^.

For maximum flux the lens position should be 10000 to 12000 mm whereas for minimum spot size the lens should be stationed at around 28000 mm. This creates conflict between the objectives. Thus, multi-objective optimization usage can be justified.

### 3.2. Multi-objective lens position optimization

**Figure 16:**
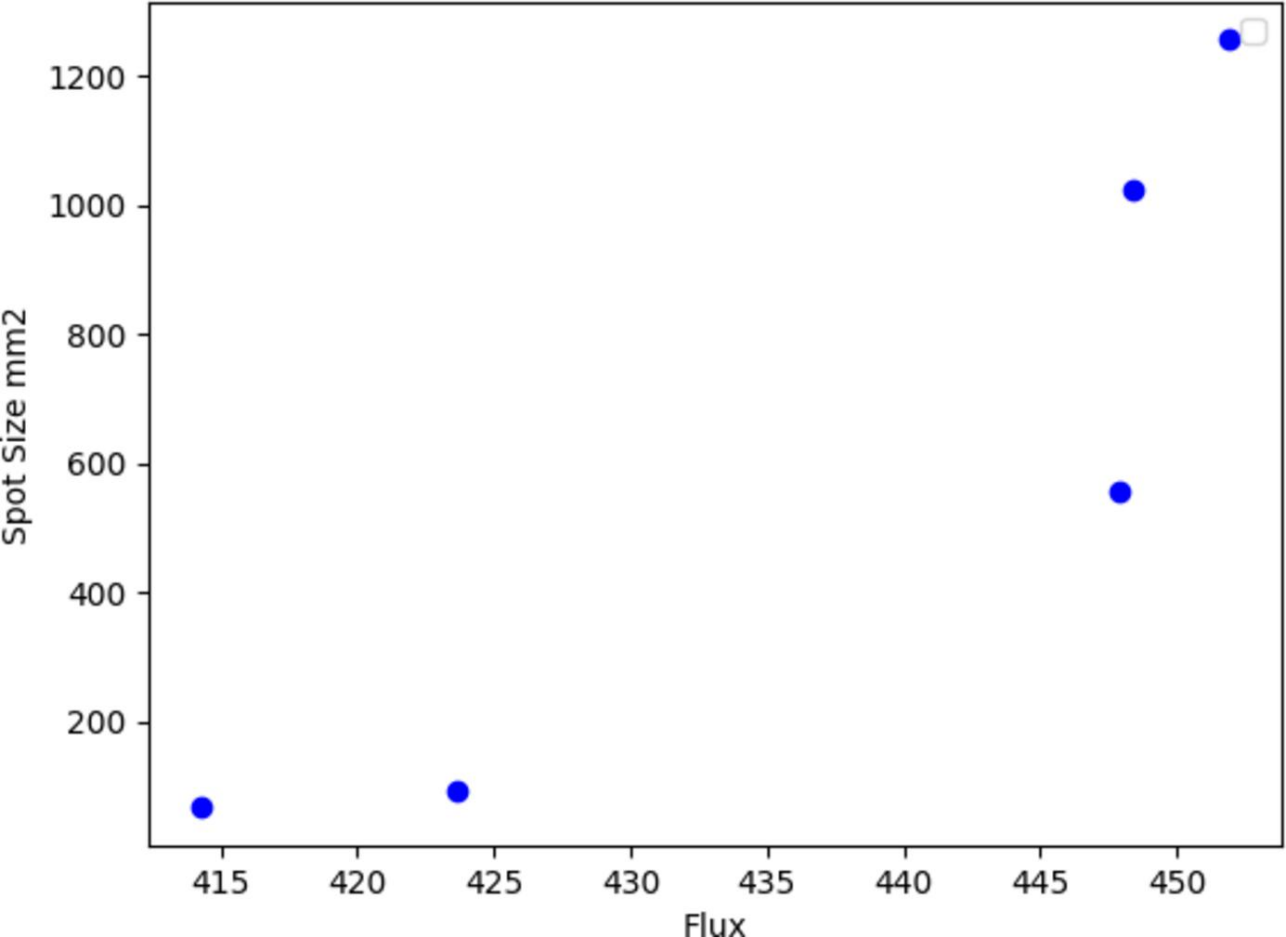
Pareto front for NSGA-II solutions.

Using NSGA-II the highest ranking solutions were selected. In Fig. 16 ▸ the desired space is in the right-hand lower corner, meaning the maximum flux and minimum spot size. After the dominated results were cleaned out, only five distances remained out of 20 since the population was set to 20 for each experiment, just like for single-objective optimization where we are only interested in the best value.

As can be seen, the results from the NSGA-II algorithm have an inverse proportion relation in Fig. 16 ▸ considering maximum flux and minimum spot size are desired which is in the lower right corner. Starting from the left the distances were found to be 17085, 28926, 29376, 16609 and 17687 mm, and some values leaned towards the minimum spot size distance which is a juxtaposition of 28000 mm. Some values are between 10000 and 28000 mm.

### 3.3. Mono-objective mirror focal distance optimization

**Figure 17:**
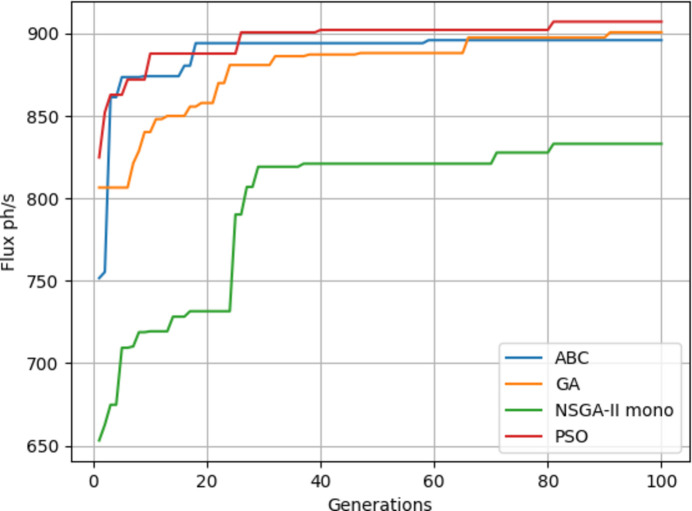
Maximum flux results.

Four algorithms were run with flux as the objective. It can be seen from Fig. 17 ▸ that NSGA-II gives a poorer performance on average while PSO gives slightly better results. The results of Fig. 17 ▸ are also given in Table 3 ▸. From Tables 4 ▸ and 5 ▸ it can be seen that the objective values are much better since KB mirrors give a better focus than when using only a lens.

Since the beam is elliptical, giving both mirrors the same or relative focal distances does not provide good results. For mono-optimization, PSO gives the best results of the four algorithms. The reason for this must be that swarm intelligence does not create solely random decision variables except the first generation. It includes local and global best solution positions, and EA operators such as mutation and crossover send some of the solutions towards unknown spaces in the search field. Thus PSO is more efficient for this type of problem. ABC is more complex than PSO so that could be the reason for the difference between their performances.

For maximum flux, NSGA-II gives the worst results. Mirrors have a mixture of focal distances, because one mirror focuses vertically while the other focuses horizontally. For spot size, the focal distance of the first mirror should be around 2000 mm which is the distance between the first mirror and sample. The focal distance of the second mirror should have huge values that make it practically flat. Since KB mirrors cannot be completely flat, the second mirror should have a slight bending. Thus the focal distance of the first mirror can never be exactly 2000 mm since this focuses the beam before the sample and beams are scattered after focusing.

**Figure 18:**
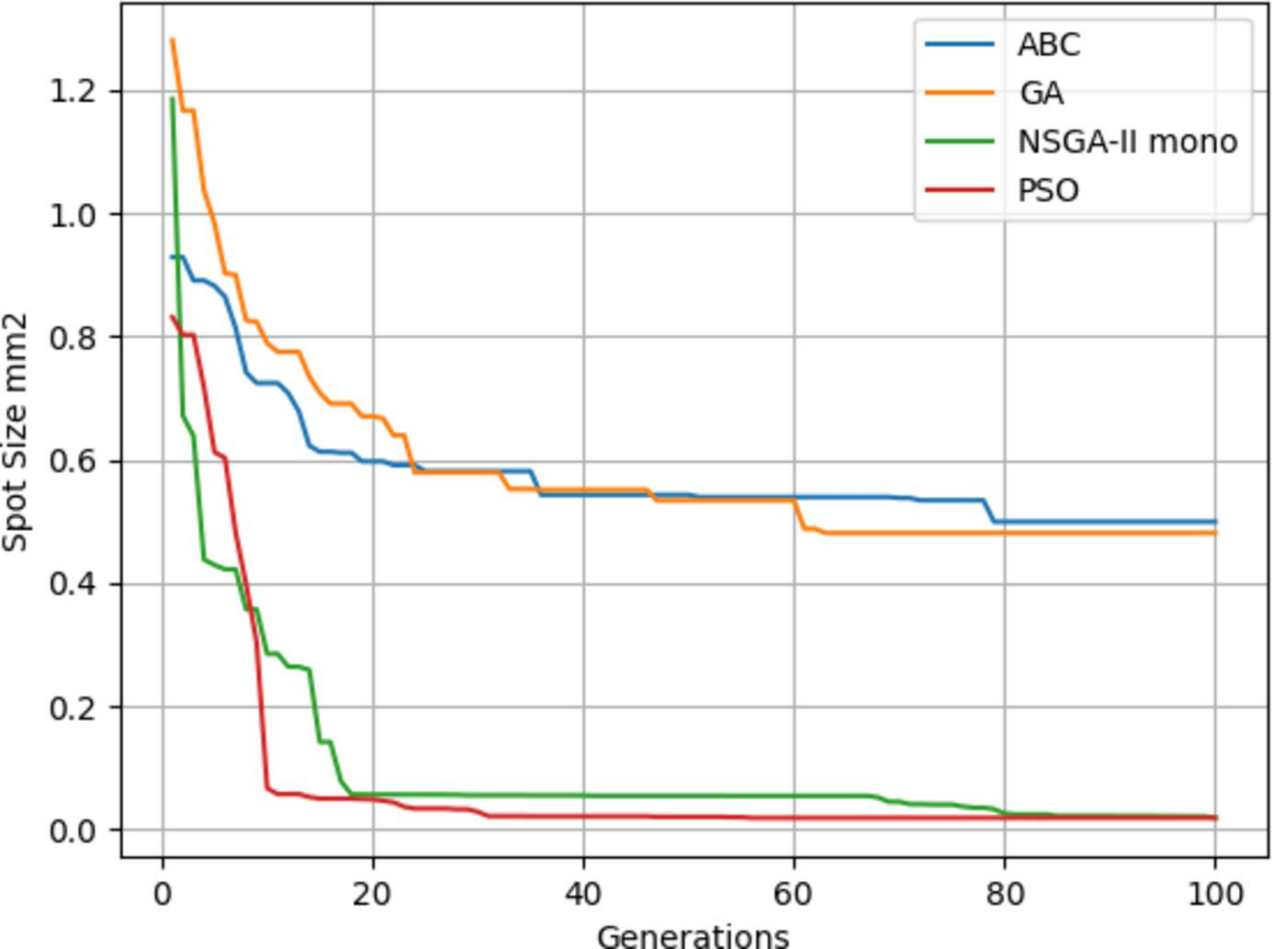
Minimum spot size results.

Fig. 18 ▸ shows the fitness through the generations. Best values are provided by PSO. Since the focal distance values are completely different for the two objectives, we are going to use multi-objective optimization.

### 3.4. Multi-objective mirror focal distance optimization

The spot size value and flux value are optimized simultaneously using the NSGA-II algorithm with two objectives. The *q*1 and *q*2 values for three runs are shown in Tables 6 ▸, 7 ▸ and 8 ▸.

As can be seen from the results, the total results are biased towards minimum spot size. Most of the *q*1 values are around 2000 mm. This is in conformity with Fig. 17 ▸ because the mono-objective results were between 833 and 907 photons s^−1^ for flux and approximately 0.02 mm^2^ for spot size. Since every run is a different stochastic process, it is safe to assume that the best value is around 900 photons s^−1^ and 0.01 mm^2^. As for the *q*2 values, it can be seen that they are larger compared with the *q*1 values.

**Figure 19:**
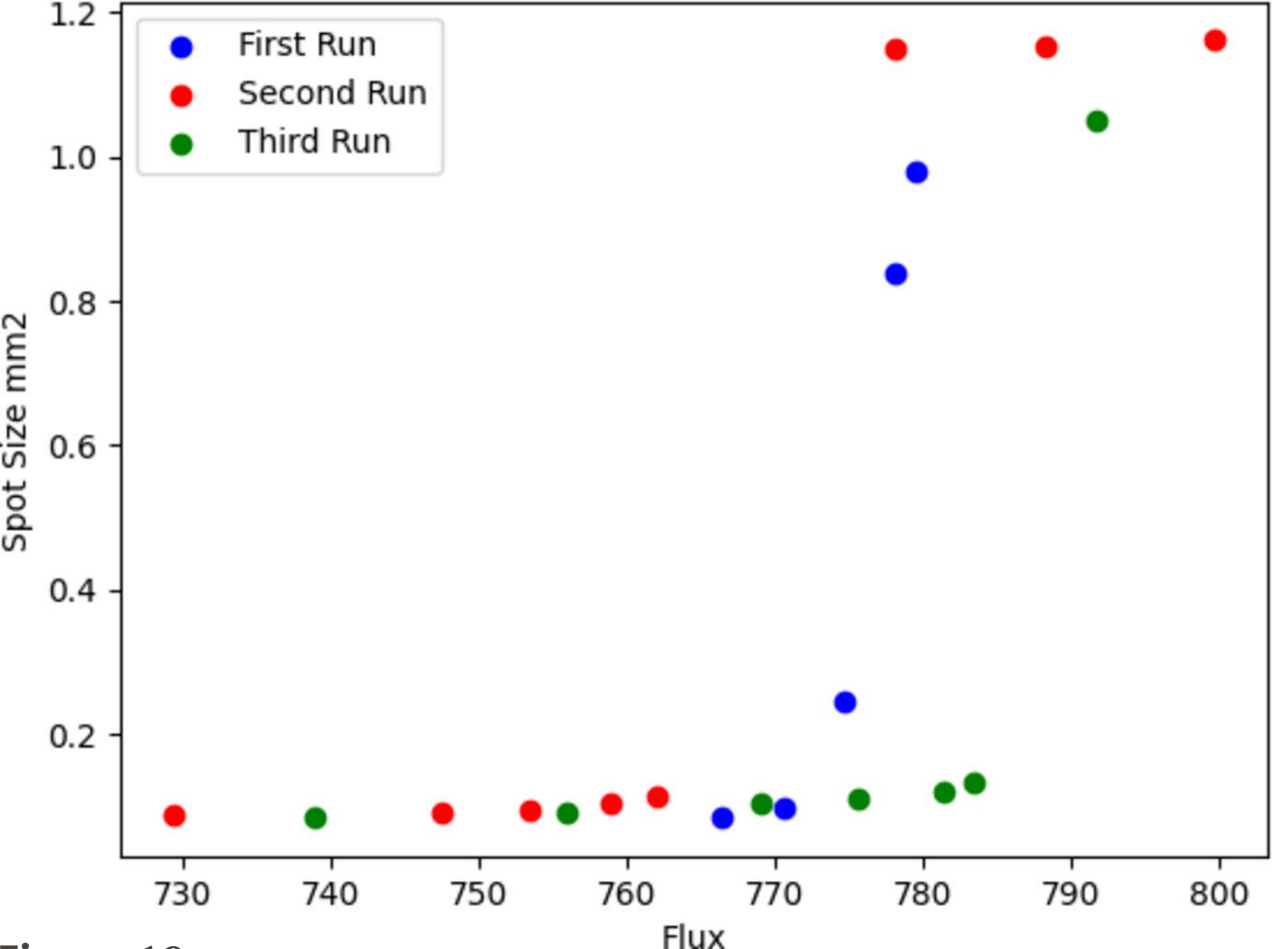
Multi-objective results for the three runs.

The Pareto fronts for every run have different element numbers as can be seen from Fig. 19 ▸; thus it is difficult to calculate the average. Individual results might dominate each other since they are independent processes. For this reason it is difficult to draw conclusions, but to the best of our ability we can infer that the results are biased towards minimum spot size where the flux values drop significantly. Calculations were carried out at 9000 eV, and 100000 rays were deployed on experiments which is quite low compared with real-life experiments. For a quicker observation of the system, the source parameters were reduced. Thus spot size and flux diverge from real-life experiment results. Nevertheless, the method should be the same for every beamline parameter.

## 4. Conclusion

For every optimization problem, some algorithms and techniques may give better performance than others. Thus different methods should be tested at first for every domain. When four algorithms (ABC, GA, NSGA-II mono and PSO) were compared on a single objective mode, PSO showed superior performance for this type of optimization problem where efficiency is increased step by step.

If we were to reflect sunlight with a plane mirror, first we need to adjust the angle until the light hits the target surface. Angles that are too narrow or too wide do not give any results. In a sense it is a scanning process. After hitting the surface, we focus the light for a better flux on a certain area. With this analogy in mind, the random jumps on EAs slow down the process whereas PSO includes the global and local best. If parameters of each algorithm were adjusted, given sufficient time any algorithm would have given the best results. But that means losing time for parameter optimization. With the same set of parameters PSO is the best choice on a single objective.

Beamline alignment is prone to errors. By adjusting the optical elements, spot size and flux values can change significantly. Just like the algorithms, the objective functions should be tested. The simulation tends to give better results on spot size when the two objective functions spot size and flux are used. Depending on the experiment type, the objective function should be determined in favor of the requirements.

Since comparison of multi-objective algorithms takes different metric calculations such as hypervolume, spacing, error ratio and inverted generational distance *etc*., it will be considered for a future study.

## 5. Data availability

The generated and/or analyzed datasets during the study are available from the corresponding author upon reasonable request.

## Figures and Tables

**Table 1:** Geometric source parameters

Electron energy	9 ± 0.5 keV
Polarization	Horizontal
d*x*′	1 × 10^−5^
d*z*′	3 × 10^−6^
Number of rays	100000
Energy distribution	Normal

**Table 2:** Maximum flux calculation results

Algorithm	Distance (mm)	Flux (photons s^−1^)
ABC	10570	476.39557
GA	11665	483.55616
NSGA-II	10188	481.49050
PSO	10095	**486.51149**

**Table 3:** Minimum spot size calculation results

Algorithm	Distance (mm)	Spot size (mm^2^)	*X*-axis (mm)	*Z*-axis (mm)
ABC	28723	55.13	13.48	53.45
GA	28869	53.24	12.26	51.82
NSGA-II	28605	54.85	14.98	52.77
PSO	28843	**51.59**	**12.11**	**50.16**

**Table 4:** Average flux objective function results and focal distances on three runs for ABC, GA, NSGA-II, PSO algorithms

	Run 1		Run 2		Run 3		
	*q*1 (mm)	*q*2 (mm)	*q*1 (mm)	*q*2 (mm)	*q*1 (mm)	*q*2 (mm)	Average flux (photons s^−1^)
ABC	29924	46223	62481	69810	37432	27147	896.01516
GA	6879	38147	28304	51984	20251	63477	900.80349
NSGA-II	25472	58603	7195	39071	49302	58113	833.13396
PSO	**17964**	**38823**	**2261**	**52871**	**22005**	**47655**	**907.22915**

**Table 5:** Average spot size objective function results and focal distances on three runs for ABC, GA, NSGA-II, PSO algorithms

	Run 1		Run 2		Run 3				
	*q*1 (mm)	*q*2 (mm)	*q*1 (mm)	*q*2 (mm)	*q*1 (mm)	*q*2 (mm)	Average spot size (mm^2^)	*X*-axis (mm)	*Z*-axis (mm)
ABC	2033	68294	2071	63869	2071	69047	0.499	0.4881	0.1027
GA	2089	62637	2058	68787	2108	69541	0.481	0.4669	0.1158
NSGA-II	2025	62692	2069	69557	2036	68609	0.0194	0.0188	0.0047
PSO	**2050**	**68247**	**2140**	**59042**	**2083**	**69326**	**0.0190**	**0.0186**	**0.0039**

**Table 6:** Multi-objective results for the first run

*q*1 (mm)	*q*2 (mm)	Spot size (mm^2^)	Flux (photons s^−1^)
2059	69503	**0.086**	766.41
26341	57142	0.979	**779.56**
1780	57572	0.245	774.75
2062	69950	0.840	778.06
2052	61890	0.097	770.66

**Table 7:** Multi-objective results for the second run

*q*1 (mm)	*q*2 (mm)	Spot size (mm^2^)	Flux (photons s^−1^)
22626	48770	1.153	788.31
12718	61341	1.161	**799.74**
2167	69770	1.151	778.06
2152	69404	0.094	753.42
2166	69846	0.091	747.54
2166	69562	**0.088**	729.31
2166	69778	0.114	761.99
2012	69404	0.105	758.86

**Table 8:** Multi-objective results for the third run

*q*1 (mm)	*q*2 (mm)	Spot size (mm^2^)	Flux (photons s^−1^)
8018	21860	1.050	**791.71**
2090	60744	0.122	781.44
2019	58713	0.133	783.48
2088	67845	**0.084**	738.91
2020	60429	0.111	775.69
2088	69157	0.106	768.99
2097	58713	0.092	755.91
